# Evaluation of the Shear Bond Strength and Antibacterial Activity of Orthodontic Adhesive Containing Silver Nanoparticle, an In-Vitro Study

**DOI:** 10.3390/nano10081466

**Published:** 2020-07-27

**Authors:** Ladan Eslamian, Ali Borzabadi-Farahani, Shahedeh Karimi, Sepideh Saadat, Mohammad Reza Badiee

**Affiliations:** 1 Dentofacial Deformities Research Center, School of Dentistry, Shahid Beheshti University of Medical Sciences, Tehran 19857-17443, Iran; leslamian@gmail.com; 2 Department of Orthodontics, School of Dentistry, Shahid Beheshti University of Medical Sciences, Tehran 19857-17443, Iran; Mohammadreza.badiee@yahoo.com; 3 Orthodontics, Department of Clinical Sciences and Translational Medicine, University of Rome Tor Vergata, 00183 Rome, Italy; 4 Private Practice, Finchley Orthodontics, North Finchley, London N12 9EN, UK; 5 School of Dentistry, Shahid Beheshti University of Medical Sciences, Tehran 19857-17443, Iran; ShahedehKarimi@yahoo.com (S.K.); SepidehSaadat@yahoo.com (S.S.)

**Keywords:** antibacterial, remineralization, shear bond strength, orthodontics, silver nanoparticles, adhesive remnant index, caries inhibition

## Abstract

This study evaluated the effect of incorporating silver nanoparticles (AgNPs) into conventional orthodontic adhesive on its antibacterial activity and the shear bond strength (SBS) to stainless steel orthodontic brackets. Thirty-four extracted premolars were randomly allocated into two groups (*n* = 17). Orthodontic adhesive (Transbond XT, 3M Unitek) was blended with AgNPs (50 nm, 0.3% *w*/*w*) to form a nano-adhesive. In order to bond stainless steel twin brackets (0.022-inch, American Orthodontics), Transbond XT (*n* = 17) and nano-adhesive (*n* = 17) were used in each group, respectively, after acid etching (37% phosphoric acid, 30 s) and rinsing with water (15 s). SBS and the adhesive remnant index (ARI) scores were recorded. Antibacterial activity against *Streptococcus mutans* in both groups after 24 h and 30 days was assessed (Disc agar diffusion test) and the inhibition zone diameter around each specimen was measured and recorded. Adding AgNPs significantly (*p* = 0.009) reduced the mean (SD) SBS in the nano-adhesive group [10.51(7.15) MPa] compared to Transbond XT [17.72(10.55) MPa]. The ARI scores on the Transbond XT and nano-adhesive showed no statistically significant difference (*p* = 0.322). Nano-adhesive with AgNPs showed significant antibacterial activity against *Streptococcus mutans* at 24 h and 30 days (*p* < 0.001). In both groups, no significant decline in the zones of inhibition was detected after 30 days (*p* = 0.907). The findings suggest that SBS decreased after incorporation of AgNPs [0.3% (*w/w*)], but was still above the recommended SBS of 5.9–7.8 MPa. The nano-adhesive showed significant antibacterial activity which did not change much after 30 days.

## 1. Introduction

Enamel demineralization or white spot lesions can occur during orthodontic treatment and can be seen in up to 96% of orthodontic patients [1,2,3,4]. This is due to hampering the maintenance of oral hygiene and provision of numerous additional surfaces for formation of bacterial biofilm by fixed orthodontic appliances [1,2,3,4]. Literature review reveals a number of recommendations to eliminate or reduce the white spot formation, such as oral hygiene education, tele-monitoring, selective etching technique, and application of fluoride, which are mainly reliant on patients’ cooperation [5,6].

Fluoride application produces a less soluble, carrying resistant fluorapatite on the enamel surface, which is more resistant to acid attack [7]. Fluoride delivery comes in different forms, such as fluoride varnishes, or toothpastes, mouth-rinses, and gels containing fluoride, as well as orthodontic bonding adhesives/cements incorporating a source of fluoride [7,8]. Other recent approaches to reduce white spot formation include the application of Casein Phosphopeptide-Amorphous Calcium Phosphate (CPP-ACP), bioactive glass, or addition of nano-particles with anti-caries activities to the orthodontic appliance surfaces or orthodontic adhesives/cements [2,8,9,10,11,12].

As outlined earlier, contemporary approaches are mainly investigating the effect of antibacterial agents incorporated into orthodontic adhesives or cements, or used for coating orthodontic appliances with them, to reduce bacterial aggregation [13] and prevent white spot formation [2].

The use of silver nanoparticles (AgNPs) in orthodontic adhesives is relatively new [2]; they have interesting properties such as chemical stability, catalytic activity, localized surface plasma resonance, and high conductivity [14,15]. We know that the reactive oxygen species form at the surface of the silver nanoparticles or by the released free silver ions which, under certain conditions, can induce cell death of either mammalian cells or microbial cells, giving the silver nanoparticles unique antibacterial and anti-fungal properties [14,15].

The objective of the present in-vitro study was to investigate the effect of addition of AgNPs (50 nm avg. part. size. 0.3% *w/w*) into an orthodontic adhesive on the shear bond strength (SBS) and adhesive remnant index (ARI) between stainless steel brackets and enamel tooth surface. The null hypothesis presumed that there were no significant differences between SBS values and bond failure sites (ARI) of stainless-steel brackets bonded to enamel tooth surfaces using conventional adhesive and nano-adhesive.

## 2. Materials and Methods

### 2.1. Nano-Adhesive Preparation

**Figure 1:**
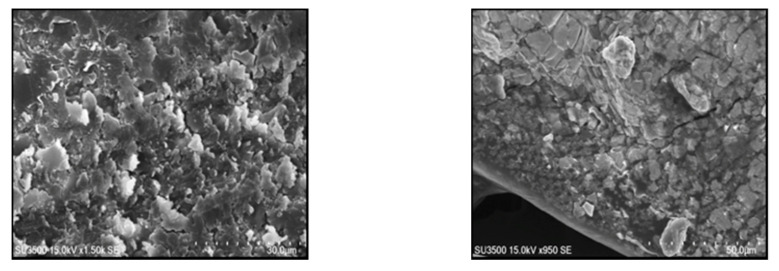
The scanning electron microscopy (SEM) view of the nano-adhesive under scanning electron microscope.

The Transbond XT (3M Unitek, CA, USA) orthodontic adhesive was used to bond the brackets. Light cure orthodontic adhesive (Transbond XT) was blended with silver nanoparticles (Sigma-Aldrich Biotechnology, St Louis, MO, USA, Silver nanospheres 50 nm avg. part. size, 0.3% *w/w*) using a mixer at 3500 rpm for 5 min in a dark room. After the preparation, the nano-adhesive was observed under scanning electron microscope to check the homogeneity of the mix (Figure 1).

### 2.2. Sample Size Calculation and Specimen Preparation

Based on the previous study [16], the minimum standardized difference, type I error rate (0.05), and power (0.80) for this investigation, 34 recently extracted non-carious, non-fluorosed, human premolars with sound buccal surfaces were selected (17 in each group). The teeth were cleaned, lightly pumiced (5 s), and stored in distilled water at room temperature before use.

For this experiment, the stainless-steel maxillary premolar twin brackets (0.022-inch, American Orthodontics, Sheboygan, WI, USA) were used as follows:

Group 1. The buccal surface was etched for 30 s with 37 per cent phosphoric acid at room temperature, rinsed for 15 s with water, and air dried till white, chalky surface appeared. Subsequently, after application of the Transbond XT primer, a light cure orthodontic adhesive (Transbond XT, 3M Unitek, CA, USA) was used to bond the stainless-steel brackets to the tooth surface. All brackets were light cured (Optilux 50; Kerr, Danbury, CT, USA, with a light intensity of 650 mW/cm^2^) for 40 s (10 s for mesial, distal, gingival and occlusal sides). Excess adhesive was removed using an explorer before light curing.

Group 2. The bonding procedure similar to group 1 was used, but the nano-adhesive was used to bond the brackets to the tooth surface.

Then, all specimens were thermocycled 500 times between 5 °C and 55 °C with a dwell time of 30 s between each cycle. Finally, they were vertically mounted in auto-polymerizing acrylic blocks with the buccal surfaces parallel to the debonding blade.

### 2.3. Assessment of Shear Bond Strength and Adhesive Remnant Index

Shear bond strength (SBS) of two groups were determined, and the adhesive remnant index (ARI) scores were also assessed.

#### 2.3.1. Shear Bond Strength

The shear bond strength was tested using a universal testing machine (Z020; Zwick GmbH, Ulm, Germany). The shear force at a crosshead speed of 1 mm/minute was transmitted to the bracket and the teeth were aligned so that the applied force was perpendicular to the bracket. The force required to shear the bracket was recorded and the bond strength was calculated in megapascals (MPa). The findings of this study were compared with the recommended bond strength of 5.9–7.8 MPa suggested by Reynolds [17].

#### 2.3.2. Adhesive Remnant Index

The sheared surfaces were further investigated with a stereomicroscope (Olympus, SZX9, Tokyo, Japan) at 20× magnification to assess the adhesive remnants on the specimen surface. The adhesive remnant index (ARI) as described by Artun and Bergland [18] was used and recorded for this assessment. ARI scores were used as a means of defining the sites of bond failure between the enamel, resin (adhesive), and the bracket base. The ARI (the substrate ARI score or ARIs) was scored 0–3, as follows:

0, no adhesive left on the tooth;
1, less than half of the adhesive left on the tooth;
2, more than half of the adhesive left on the tooth;
3, all the adhesive left on the tooth with the mesh pattern visible.

### 2.4. Determination of the Antibacterial Activity of the Silver Nanoparticles (AgNPs)

For this experiment, 68 composite disk (6 mm diameter and 3 mm thickness, 34 nano-adhesive and 34 conventional adhesive (Transbond XT) disks) were fabricated using plastic molds. Molds were covered by on each side with matrix strips and light cured on each side for 20 s. There was no distance between the LED light tip and the top surface of the composite specimen. Then, specimens were exposed to UV light (15 min) to make sure there is no contamination. Each specimen group were further divided into two groups of 17 (17 nano-adhesive and 17 conventional adhesive disks) and used for disc agar diffusion test to assess the antimicrobial activity after 24 h (*n* = 34) and after 30 days (*n* = 34).

#### Disc Agar Diffusion Test

Disc agar diffusion test was used to assess the antibacterial effects of adhesive containing Silver nanospheres (50 nm avg. part. size, 0.3% *w/w*) against S. mutans (PTCC 1683, purchased from the Iranian Research Organization for Science and Technology (IROST)). A McFarland 0.5 bacterial suspension was used, using sterile swaps, and was cultured on agar plates and immediately exposed to discs from each group (*n* = 34). The plates were incubated for 24 h at 37 °C and the diameter of bacterial growth inhibitory zone around the discs was measured in mm. This procedure was repeated after 30 days on the other 34 specimens that were kept in normal saline for 30 days to assess the longitudinal changes in the diameter of bacterial growth inhibitory zone.

## 3. Statistical Analysis

Statistical analysis was performed with SPSS software, Version 17 (Statistical Package for Social Sciences; SPSS Inc., Chicago, IL, USA). The data for the SBS were subjected to the Shapiro–Wilk normality test and revealed that data was not normally distributed (*p* < 0.05), and therefore, the non-parametric Mann–Whitney test was used to analyze the SBS data. For the comparison of the ARI scores, the Mann–Whitney test was also used.

The data for inhibition zone diameter was exposed to normality test (*p* > 0.05) and then compared with two-way ANOVA and Tukey post-hoc test. The significance level was (*p* < 0.05).

## 4. Results

### 4.1. Shear Bond Strength

The Mann–Whitney test showed that there was a significant SBS difference (*U* = 69, *p* = 0.009) between the nano-adhesive group with silver nanoparticles [10.51 (7.15) MPa, range: 4.19–29.17 MPa] and the Transbond XT orthodontic adhesive group [17.72 (10.55), MPa, range: 6.10–40.31 MPa], but this was still above the clinically recommended SBS of 5.9–7.8 MPa.

### 4.2. Adhesive Remnant Index

The amount of residual adhesive on the specimen surfaces as evaluated by the ARI scores is shown in Table 1. The ARI scores for the Transbond XT orthodontic adhesive and nano-adhesive showed no statistically significant differences (The Mann–Whitney test, *U* = 115.5, *p* = 0.322). Overall, the null hypothesis was partially rejected (SBS) for this study.

### 4.3. Antibacterial Activity

**Figure 2:**
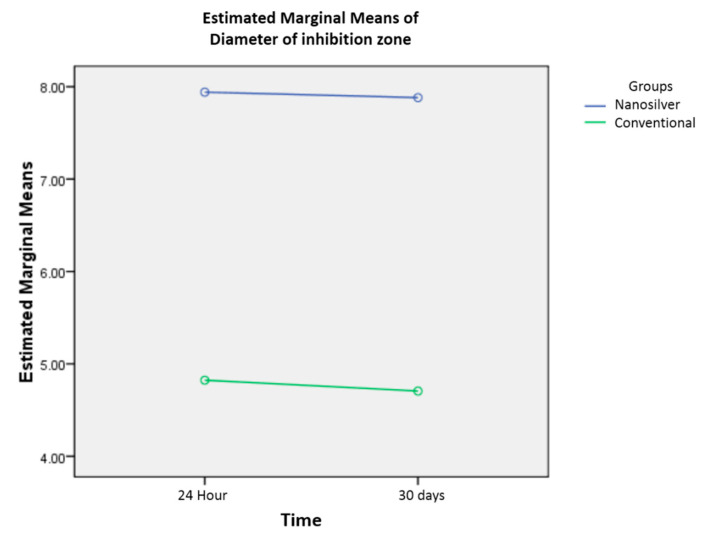
Inhibition zone diameter for conventional adhesive and nano-adhesive at 24 h and 30 days (mm). No interaction effect can be seen as there is a set of nearly parallel lines for both conventional adhesive and nano-adhesive.

The mean (SD) of the inhibition zone diameter for conventional adhesive at 24 h and 30 days were 4.82 (1.18) and 4.70 (0.91) mm, retrospectively. The figures for the nano-adhesive (AgNPs) group were 7.94 (0.96) and 7.88 (1.05) mm, retrospectively. The inhibition zone diameter was significantly larger in the nano-adhesive group at 24 h and 30 days (*p* < 0.001). In both groups, there was no significant interaction in inhibition zone diameter at 24 h and 30 days (F (1, 64) = 0.014, *p* = 0.907) (Figure 2).

## 5. Discussion

Incorporation of nanoparticles (NPs) into orthodontic material has revealed promising capacities in terms of antimicrobial and mechanical properties [2], but needs further investigation [19]. NPs have been added to orthodontic adhesives, cements, or acrylic resins and can be coated onto the surfaces of orthodontic appliances to prevent microbial adhesion or enamel demineralization during orthodontic treatment [2]. Silver (Ag) has antibacterial, anti-fungal and antiviral properties [20,21]. Resins containing NPs of silver were synthesized that have antibacterial characteristics [22,23,24]. These properties are mainly due to the release of Ag^+^ ions, which is higher when fine AgNPs are used (<10 nm particle size) for antibacterial action compared to larger AgNPs [25]. Overall, they can cause cell leakage, destabilization of ribosomes, enzyme interaction, production of reactive oxygen species, DNA damage, and cell death [25].

There are a few in-vitro studies that investigated the effect of incorporation of AgNPs into orthodontic adhesives [26,27,28,29,30,31]. Ahn et al. reported that experimental adhesives composed of 250 ppm and 500 ppm AgNPs produced interesting antibacterial properties without adversely affecting the shear bond strength [26]. Blöcher et al. [27] examined the effect of addition of micro-silver particles (0.1% and 0.3% (*w/w*) with particle sizes of 3.5–18 μm) and AgNPs particles (0.11%, 0.18% and 0.33% (*w/w*) with particle sizes of 12.6–18.5 nm) to Transbond XT primer and reported no significant effect on SBS or ARI scores of the experimental adhesive to the bovine incisor teeth. However, the type of specimens used [27] or the concentrations of the AgNPs were different, compared to the present experimental setting [26]. Similar to the present findings, Reddy et al. incorporated 1% AgNPs to the orthodontic adhesive (Transbond) and reported significant reduction in SBS [28]. Addition of 0.11%, 0.18%, and 0.33% (*w/w*) of AgNPs the orthodontic adhesive (Transbond) primer similarly reduced the SBS of the experimental adhesive [29]. Another experiment by Riad et al. also reported reduced SBS of the experimental adhesive containing AgNPs [30].

Reynolds [17] recommended minimum bond strength of 5.9–7.8 MPa as adequate bond strength for most orthodontic needs during routine clinical use. The bond strength value of the nano-adhesive used in the present study was above this minimum requirement and offered clinically acceptable mean SBS, but with a wide range, suggesting further clinical investigations. This is in line with recent systemic review findings suggesting that mild reduction in SBS of orthodontic adhesives is expected after additions of NPs [32]. However, clinical conditions may differ significantly compared to an in-vitro setting and therefore, further clinical studies are needed. We know from previous research that AgNPs are effective against *Streptococcus mutans*, a major contributor to dental cavities, a higher antimicrobial effect against *Streptococcus mutans* of AgNPs at lower concentrations compared to gold or zinc nanoparticles, allowing important clinical effects with a reduced toxicity [33]. We used 0.3% weight concentrations of AgNPs and measured the diameter of growth inhibition at 24 h and 30 days observing significant antibacterial effect at both time points. Previous studies confirmed that orthodontic adhesive with AgNPs were effective against *Streptococcus mutans* [34,35]; however, some did not report the same effect after 30 days. For 0.5% and 1% weight concentrations [34], although there was difference in the diameter of growth inhibition at 24 h, the same effect was not observed at 30 days. Therefore, authors suggested a short-term antibacterial effect for AgNPs [34]. The concentration used in the present study (0.3% weight) was lower and differences in methodology may explain the different outcome at 30 days. Another study [29] used 0%, 0.11%, 0.18%, and 0.33% weight concentrations of AgNPs; after 48 h, they reported growth inhibition of S. mutans in liquid media after silver addition; however, no inhibitory halos (Disk diffusion assay) were detected around disks of any silver concentration [29]. Orthodontic adhesive discs containing 5 and 10 % silver/hydroxyapatite nanoparticles produce bacterial growth inhibition zones and show antibacterial properties against biofilms, but the same effect was not observed when 1% silver/hydroxyapatite nanoparticles was added to the orthodontic adhesive [31].

Recent studies suggest a combination of antimicrobial or protein-repellent material and calcium phosphate nanoparticle to remineralize the carious lesion. The cytotoxicity and biocompatibility are important considerations for the new bioactive materials. In another word, rather than killing specific bacteria, it is more desirable to modulate the oral biofilm compositions via bioactive resins to suppress cariogenic/pathogenic species and promote benign species [35]. However, there is no standard protocol for incorporation of AgNPs into the orthodontic adhesives, such as optimal *w*/*w* concentration of NPs or addition to adhesive or primer; almost all studies are in-vitro studies [2]. There is a need for long-term clinical trials to investigate the clinical performance and risk/benefits of incorporating AgNPs for improving anti-caries properties of orthodontic adhesives. These should identify the right concentration/particle size of AgNPs and form of preparation (addition to primer vs. adhesive).

## 6. Conclusions

The in-vitro findings suggest that shear bond strength decreased after incorporation of AgNPs (0.3% (*w/w*)), but was still above the recommended SBS of 5.9–7.8 MPa. This was associated with significant antibacterial activity did not change much after 30 days. Long-term clinical trials are needed to examine the clinical performance and risk/benefits of incorporating AgNPs for improving anti-caries properties of orthodontic adhesives. These should identify the right concentration/particle size of AgNPs and form of preparation (addition to primer vs. adhesive).

## Figures and Tables

**Table 1:** The adhesive remnant index (ARI) scores on enamel tooth surfaces in 2 groups.

	ARI				Total
	0	1	2	3	
Transbond XT	1 (5.9)	5 (29.4)	10 (58.8)	1 (5.9)	17
Nano-adhesive	0	4 (23.5)	10 (58.8)	3 (17.6)	17
Total	1 (2.9)	9 (26.5)	20 (58.8)	4 (11.8)	34

## References

[1] Bishara S.E., Ostby A.W. (2008). White Spot Lesions: Formation, Prevention, and Treatment. Semin. Orthod..

[2] Borzabadi-Farahani A., Borzabadi E., Lynch E. (2013). Nanoparticles in orthodontics, a review of antimicrobial and anti-caries applications. Acta Odontol. Scand..

[3] Chapman J.A., Roberts W.E., Eckert G.J., Kula K., González-Cabezas C. (2010). Risk factors for incidence and severity of white spot lesions during treatment with fixed orthodontic appliances. Am. J. Orthod. Dentofac. Orthop..

[4] Ren Y., Jongsma M.A., Mei L., Ren Q., Busscher H.J. (2014). Orthodontic treatment with fixed appliances and biofilm formation—a potential public health threat?. Clin. Oral Investig..

[5] Zotti F., Dalessandri D., Salgarello S., Piancino M., Bonetti S., Visconti L., Paganelli C. (2015). Usefulness of an app in improving oral hygiene compliance in adolescent orthodontic patients. Angle Orthod..

[6] Dalessandri D., Dalessandri M., Bonetti S., Visconti L., Paganelli C. (2012). Effectiveness of an indirect bonding technique in reducing plaque accumulation around braces. Angle Orthod..

[7] Chambers C., Stewart S., Su B., Sandy J., Ireland A. (2013). Prevention and treatment of demineralisation during fixed appliance therapy: A review of current methods and future applications. Br. Dent. J..

[8] Taha A.A., Patel M.P., Hill R.G., Fleming P.S. (2017). The effect of bioactive glasses on enamel remineralization: A systematic review. J. Dent..

[9] Cochrane N., Reynolds E.C. (2012). Calcium Phosphopeptides—Mechanisms of Action and Evidence for Clinical Efficacy. Adv. Dent. Res..

[10] Cochrane N., Saranathan S., Cai F., Cross K.J., Reynolds E.C. (2008). Enamel Subsurface Lesion Remineralisation with Casein Phosphopeptide Stabilised Solutions of Calcium, Phosphate and Fluoride. Caries Res..

[11] Beerens M.W., Cate J.M.T., Buijs M.J., Van Der Veen M.H. (2017). Long-term remineralizing effect of MI Paste Plus on regression of early caries after orthodontic fixed appliance treatment: A 12-month follow-up randomized controlled trial. Eur. J. Orthod..

[12] De Almeida C.M., Da Rosa W.L.O., Meereis C.T.W., Ribeiro J.S., Da Silva A.F., Lund R.G., De Almeida S.M. (2018). Efficacy of antimicrobial agents incorporated in orthodontic bonding systems: A systematic review and meta-analysis. J. Orthod..

[13] Ghasemi T., Arash V., Rabiee S.M., Rajabnia R., Pourzare A., Rakhshan V., Rabiee M., Nia R.R., Zadeh F.M., Pour Zare A.H. (2017). Antimicrobial effect, frictional resistance, and surface roughness of stainless steel orthodontic brackets coated with nanofilms of silver and titanium oxide: A preliminary study. Microsc. Res. Tech..

[14] Wang M., Marepally S., Vemula P., Xu C. (2016). Inorganic Nanoparticles for Transdermal Drug Delivery and Topical Application. Nanoscience in Dermatology.

[15] Tran Q.H., Le A.T. (2013). Silver nanoparticles: Synthesis, properties, toxicology, applications and perspectives. Adv. Nat. Sci. Nanosci. Nanotechnol..

[16] Poosti M., Ramazanzadeh B., Zebarjad S.M., Javadzadeh P., Naderinasab M., Shakeri M.T. (2012). Shear bond strength and antibacterial effects of orthodontic composite containing TiO_2_ nanoparticles. Eur. J. Orthod..

[17] Reynolds I.R. (1975). A Review of Direct Orthodontic Bonding. Br. J. Orthod..

[18] Artun J., Bergland S. (1984). Clinical trials with crystal growth conditioning as an alternative to acid-etch enamel pre-treatment. Am. J. Orthod..

[19] Levard C., Hotze E.M., Lowry G.V., Brown G.E. (2012). Environmental Transformations of Silver Nanoparticles: Impact on Stability and Toxicity. Environ. Sci. Technol..

[20] Monteiro D.R., Gorup L.F., Takamiya A.S., Ruvollo-Filho A.C., Camargo E.R., Barbosa D.B. (2009). The growing importance of materials that prevent microbial adhesion: Antimicrobial effect of medical devices containing silver. Int. J. Antimicrob. Agents.

[21] Allaker R.P. (2010). The Use of Nanoparticles to Control Oral Biofilm Formation. J. Dent. Res..

[22] Cheng L., Weir M.D., Xu H.H.K., Antonucci J.M., Kraigsley A.M., Lin N.J., Lin-Gibson S., Zhou X. (2012). Antibacterial amorphous calcium phosphate nanocomposites with a quaternary ammonium dimethacrylate and silver nanoparticles. Dent. Mater..

[23] Fan C., Chu L., Ralph R.H., Norling B., Cardenas H.L., Whang K. (2011). Development of an antimicrobial resin—A pilot study. Dent. Mater..

[24] Zhang K., Melo M.A., Cheng L., Weir M.D., Bai Y., Xu H.H. (2012). Effect of quaternary ammonium and silver nanoparticle-containing adhesives on dentin bond strength and dental plaque microcosm biofilms. Dent. Mater..

[25] Bapat R.A., Chaubal T.V., Joshi C.P., Bapat P.R., Choudhury H., Pandey M., Gorain B., Kesharwani P. (2018). An overview of application of silver nanoparticles for biomaterials in dentistry. Mater. Sci. Eng. C.

[26] Ahn S.-J., Lee S.-J., Kook J.-K., Lim B.-S. (2009). Experimental antimicrobial orthodontic adhesives using nanofillers and silver nanoparticles. Dent. Mater..

[27] Blöcher S., Frankenberger R., Hellak A., Schauseil M., Roggendorf M.J., Korbmacher-Steiner H.M. (2015). Effect on enamel shear bond strength of adding microsilver and nanosilver particles to the primer of an orthodontic adhesive. BMC Oral Health.

[28] Patil S.R., Reddy A.K., Kambalyal P.B., Vankhre M., Khan M.Y.A., Kumar T.R. (2016). Comparative evaluation and influence on shear bond strength of incorporating silver, zinc oxide, and titanium dioxide nanoparticles in orthodontic adhesive. J. Orthod. Sci..

[29] DeGrazia F.W., Leitune V.C.B., Garcia I.M., Arthur R.A., Samuel S.M.W., Collares F.M. (2016). Effect of silver nanoparticles on the physicochemical and antimicrobial properties of an orthodontic adhesive. J. Appl. Oral Sci..

[30] Riad M., Harhash A.Y., Elhiny O.A., Salem G.A. (2015). Evaluation of the shear bond strength of orthodontic adhesive system containing antimicrobial silver nano particles on bonding of metal brackets to enamel. Life Sci. J..

[31] Sodagar A., Akhavan A., Hashemi E., Arab S., Pourhajibagher M., Sodagar K., Kharrazifard M.J., Bahador A. (2016). Evaluation of the antibacterial activity of a conventional orthodontic composite containing silver/hydroxyapatite nanoparticles. Prog. Orthod..

[32] Pourhajibagher M., Sodagar A., Bahador A. (2020). An in vitro evaluation of the effects of nanoparticles on shear bond strength and antimicrobial properties of orthodontic adhesives: A systematic review and meta-analysis study. Int. Orthod..

[33] Hernández-Sierra J.F., Ruiz F., Pena D.C.C., Martínez-Gutiérrez F., Martínez A.E., Pozos-Guillen A., Tapia-Pérez H., Castañón G.A.M. (2008). The antimicrobial sensitivity of *Streptococcus mutans* to nanoparticles of silver, zinc oxide, and gold. Nanomed. Nanotechnol. Boil. Med..

[34] Yassaei S., Nasr A., Zandi H., Motallaei M.N. (2020). Comparison of antibacterial effects of orthodontic composites containing different nanoparticles on *Streptococcus mutans* at different times. Dent. Press J. Orthod..

[35] Cheng L., Zhang K., Zhang N., Melo M.A.S., Weir M., Zhou X., Bai Y., Reynolds M., Xu H.H. (2017). Developing a New Generation of Antimicrobial and Bioactive Dental Resins. J. Dent. Res..

